# The Upper Motor Neuron—Improved Knowledge from ALS and Related Clinical Disorders

**DOI:** 10.3390/brainsci11080958

**Published:** 2021-07-21

**Authors:** Parvathi Menon, Steve Vucic

**Affiliations:** Brain and Nerve Research Centre, University of Sydney, Sydney 2139, Australia; steve.vucic@sydney.edu.au

**Keywords:** upper motor neuron, ALS, PLS, HSP, PPMS

## Abstract

Upper motor neuron (UMN) is a term traditionally used for the corticospinal or pyramidal tract neuron synapsing with the lower motor neuron (LMN) in the anterior horns of the spinal cord. The upper motor neuron controls resting muscle tone and helps initiate voluntary movement of the musculoskeletal system by pathways which are not completely understood. Dysfunction of the upper motor neuron causes the classical clinical signs of spasticity, weakness, brisk tendon reflexes and extensor plantar response, which are associated with clinically well-recognised, inherited and acquired disorders of the nervous system. Understanding the pathophysiology of motor system dysfunction in neurological disease has helped promote a greater understanding of the motor system and its complex cortical connections. This review will focus on the pathophysiology underlying progressive dysfunction of the UMN in amyotrophic lateral sclerosis and three other related adult-onset, progressive neurological disorders with prominent UMN signs, namely, primary lateral sclerosis, hereditary spastic paraplegia and primary progressive multiple sclerosis, to help promote better understanding of the human motor system and, by extension, related cortical systems.

## 1. Introduction

Upper motor neuron (UMN) is the term traditionally used for motor neurons within the central nervous system proximal to the spinal motor neuron. Dysfunction of the upper motor neuron in neurological diseases manifests with well-recognised clinical features, including (i) increased muscle tone at rest and in response to passive velocity-dependent muscle stretch [1], (ii) pyramidal pattern muscle weakness with greater dysfunction of antigravity muscle groups (flexor group in the upper limbs and the extensor group in the lower limbs) [2], (iii) exaggerated deep tendon reflexes and (iv) extensor plantar responses (the Babinski sign) [1,2].

Dysfunction of upper motor neurons is well recognised in several inherited and acquired neurological diseases. Inherited disease with prominent clinical features of UMN dysfunction include hereditary spastic paraplegia, leukodystrophies, such as Alexander’s and Krabbe disease, and craniovertebral junction anomaly. Acquired disease of the central nervous system with prominent features of UMN dysfunction include neurodegenerative disorders such as amyotrophic lateral sclerosis (ALS) and primary lateral sclerosis (PLS), inflammatory disorders such as primary progressive multiple sclerosis (PPMS) and paraneoplastic encephalitis, chronic infection with human T lymphotrophic virus-1 (HTLV)-1 and syphilis, cerebrovascular events such as ischemic and haemorrhagic stroke or cerebral palsy and rare spinal cord ischemia due to vascular malformation.

The pathophysiology of neurological diseases where UMN dysfunction predominates may yield novel insights into the complexity and division of function of the pathways that subserve motor control [3,4], including the brainstem ventromedial reticulospinal, tectospinal and vestibulospinal pathways and dorsolateral rubrospinal pathways of motor outflow. The present review will focus on the role of UMN dysfunction in the following neurological diseases: amyotrophic lateral sclerosis (ALS), primary lateral sclerosis (PLS), hereditary spastic paraplegia (HSP) and primary progressive multiple sclerosis (PPMS). Specifically, the review will summarise the clinical features, neuropathology, neuroimaging and cortical neurophysiology in ALS and UMN predominant mimicking disorders (PLS, HSP and PPMS) to obtain a better understanding of UMN function from the pathogenesis of these neurological diseases.

## 2. Amyotrophic Lateral Sclerosis (ALS)

Amyotrophic lateral sclerosis is a rapidly progressive neurodegenerative disorder of the human nervous system, exhibiting prominent features of upper motor neuron dysfunction [5]. The term ALS is used for the typical syndrome of progressive muscle weakness, wasting and fasciculations associated with upper motor neuron signs in one or more body regions [5]. The hypothesis suggesting onset of pathophysiology in the corticomotor neurons [6] in ALS was later supported by neurophysiological findings of cortical hyperexcitability as an early and specific feature of ALS [7], preceding the clinical onset of familial ALS [8] and associated with patterns of disease spread [9,10] (Table 1).

Amyotrophic lateral sclerosis is characterised by preferential patterns of muscle wasting and weakness. Specifically, the split hand [11], split hand plus [12] and split elbow [13] phenomena exhibit preferential dysfunction of muscles involved in skilled hand function or producing antigravity movement. While the mechanism for dissociated patterns of muscle weakness and wasting in ALS remain to be fully elucidated, cortical mechanisms have been supported by the finding of greater levels of cortical hyperexcitability when recording from affected muscles [14,15]. Specifically, cortical excitability studies performed on the ‘thenar group’ and hypothenar muscles in ALS reveal a greater reduction in short-interval intracortical inhibition (SICI) [16], a biomarker of cortical excitability, over the preferentially affected ‘thenar group’ of muscles. Preferential wasting of the ‘thenar group’ of muscles in ALS, namely the abductor pollicis brevis (APB) and first dorsal interosseous (FDI), when compared with the hypothenar abductor digiti minimi (ADM) muscle [14], creates the clinically well-recognised split-hand sign in ALS [11].

Clinical UMN signs are variably manifest in ALS phenotypes. The extensor plantar response is evident in approximately half of ALS patients [17], while brisk tendon reflexes and spasticity are not an invariable finding in ALS. Although upper motor neuron signs may be absent or difficult to elicit in atypical ALS phenotypes (such as flail arm variant ALS) or advanced ALS [2,18,19] subclinical UMN dysfunction has been reported [20]. Masking of upper motor neuron signs in ALS may be attributed to concurrent dysfunction of spinal motor neurons and interneurons required for manifestation of classical UMN signs. However, an interruption of the physiological basis for release of spinal motor neurons following disruption of upper motor neuron pathways also needs to be considered [2]. Specifically, dysfunction of descending excitatory propriospinal projections, reduced corticospinal inhibitory projections and reduced interneuronal and presynaptic inhibition may contribute to variable manifestation of clinical upper motor neuron signs in ALS.

### 2.1. Histopathology in ALS

Despite varying ALS phenotypes [21], the histopathology appears uniform [22] with inclusion of phosphorylated transactive response DNA-binding protein (TDP-43) in 97% of sporadic ALS patients and most familial ALS cohorts [23]. Other protein aggregations in familial ALS include misfolded superoxide dismutase 1 (SOD1) protein in patients with the SOD-1 gene mutation [24], and fused in sarcoma (FUS) protein, which disrupts the function of a variety of RNA-binding proteins and mRNA [25]. Importantly, in the p-TDP proteinopathy group of ALS, the distribution of the p-TDP occurs not only in neurons but in support cells, namely the oligodendroglia. Further, p-TDP appears to spread in the neocortex and brainstem in a phased manner, as noted in other neurodegenerative diseases [26,27]. Genetic ALS with the C9orf 72 gene mutation revealed a greater burden of pTDP inclusions at each neuropathological stage [23]. However, as all tissue in histopathology studies was from patients deceased from ALS, a disconnect between clinical disease severity and neuropathological staging needs to be considered.

### 2.2. Neuroimaging for Assessment of the Upper Motor Neuron in ALS

The initial utility of imaging in neurological disease of the brain and spinal cord was for structural assessment of tissue, which was clinically difficult to visualise. Brain and spinal cord imaging in ALS diagnosis was used for exclusion of other disorders with characteristic imaging features [5]. Though standard magnetic resonance imaging (MRI) did show a high T2 signal in the corticospinal tracts, the finding was not very sensitive or disease specific [28,29]. Advances in structural MRI techniques, specifically voxel-based morphometry (VBM), revealed grey and white matter atrophy [30,31] in ALS, though correlation with clinical phenotype has been limited. The technique of diffusion tensor imaging (DTI), which utilizes the sensitivity of MRI to magnitude and directionality of water movement, identified signal change in the corticospinal tract which correlates with disease severity [32]. DTI also identified signal changes in the corpus callosum which, however, did not correlate with clinical signs of UMN dysfunction [33].

Reconstructing fibre pathways using tractography, detecting metabolite markers of neuronal integrity or cell turn-over using magnetic resonance spectroscopy (MRS), assessing cortical networks using resting-state functional MRI (R-fMRI) techniques and assessing grey matter perfusion with arterial spin labelling (ASL) are newer technologies being developed to assess functional changes in the upper motor neuron in disease [34] which may precede structural measurements and may help develop sensitive diagnostic and prognostic ALS biomarkers.

### 2.3. Neurophysiological Assessment of the Upper Motor Neuron in ALS

The technique of electrical stimulation of the motor cortex to record motor evoked potentials in target upper limb muscles was developed [35] in the early 20th century. However, it is the better-tolerated technique of transcranial magnetic stimulation (TMS) [36] which has been utilized extensively to study neurophysiological changes in the motor cortex in ALS [37]. Advances in neurophysiology techniques have helped develop a better understanding of UMN pathophysiology [37] and to identify potential markers of diseases [7], specifically in ALS.

A consistently reported neurophysiological abnormality in ALS, is the phenomenon of cortical hyper excitability thought to result from a loss of inhibitory pathways mediated via GABA-A receptors [38,39,40]. However, cortical hyperexcitability is reduced with riluzole, an antagonist of the traditional excitatory cortical neurotransmitter, glutamate. [41,42]. These opposing findings are reconciled by recent studies which suggest an alternate hypothesis of cortical hyperexcitability in ALS, resulting perhaps from an altered balance between inhibitory and excitatory pathways [43].

Several other neurophysiological measures also provide insights into upper motor neuron pathology in ALS. The motor-evoked potential (MEP) amplitude reflects a summation of complex corticospinal volleys [44] and the density of corticomotorneuronal projections to the spinal motor neuron [45]. The MEP amplitude is suppressed by GABAergic pathways through the GABA_A_ receptors and enhanced by glutamatergic and noradrenergic pathways [46]. An increase in MEP amplitude was reported in sporadic and familial ALS [8,47] early in the disease course and may reflect cortical hyperexcitability in ALS.

Central motor conduction time (CMCT) represents the time from stimulation of the motor cortex to the arrival of the corticospinal volley at the spinal motor neuron [48]. CMCT is typically modestly prolonged in ALS [49] and may reflect the loss of fast-conducting corticomotorneuronal fibres [50] and increased desynchronization of corticomotorneuronal volleys secondary to axonal loss [51].

Cortical silent period (CSP) designates the interruption in EMG generated by voluntary motor activity in a target muscle following stimulation of the contralateral motor cortex. CSP is mediated by spinal mechanisms in its early part and cortical inhibitory mechanisms acting via GABA_B_ receptors in the latter part [52]. Reduction in CSP, most prominent in early disease, was reported in familial and sporadic ALS [8,39,53] and is thought to reflect reduced cortical GABAergic inhibition.

The motor threshold (MT) reflects the ease of stimulation of the corticomotor neuron and reflects the density of projection of corticomotorneurons on the spinal motor neuron [37,54]. MT is reduced by glutamatergic activity [55] and raised by voltage-gated sodium channel blockade [56]. Variable changes in MT have been reported in ALS [47,57] and may reflect differences in ALS phenotypes, disease duration and disease progression at the time of testing [37].

## 3. Primary Lateral Sclerosis (PLS)

Primary Lateral Sclerosis (PLS) is an adult-onset, sporadic, slowly progressive neurodegenerative disorder of the upper motor neuron. The term is attributed to Charcot [58], who described the sclerosis of the lateral columns of the spinal cord in a woman deceased with limb contractures in an era before the concept of upper and lower motor neuron was formalised. PLS has a debated positioning on the clinical and pathophysiological spectrum of ALS. Current diagnostic criteria are clinical and require exclusion of mimic disorders. Diagnostic certainty increases with prolonged duration of a UMN-limited disorder [59].

Demographically, PLS is a non-familial disorder with slight male preponderance, low incidence [60,61] and slow progression, with average disease duration being 7.9 years or longer [62]. The age of onset of PLS has been noted to be earlier than classical ALS but later than hereditary spastic paraparesis. The onset of PLS is insidious, with the commonest site of onset being the lower limbs and the second most common region of involvement reported to be bulbar. Bulbar involvement in PLS is reportedly associated with late manifestation of emotional lability; the so-called pseudobulbar affect [63]. In classical PLS, lower motor neuron loss is not evident clinically by way of muscle wasting or weakness or detected neurophysiologically as denervation changes in the muscles [59]. Upper limbs are noted to have preserved strength despite brisk reflexes. Dementia with frontal lobe features was reported to be a late and infrequent feature of the syndrome; however, this has been debated in recent studies [64]. Uncommon clinical manifestations include gaze palsies of smooth pursuit or saccades and bladder dysfunction by way of urgency and incontinence, both of which are late and infrequent clinical manifestations. The earlier description of PLS being a pure upper motor neuron system disorder now seems less likely [64,65] with other cortical systems also seen to be involved (Table 1).

### 3.1. Histopathology in PLS

Histopathology in PLS has been obtained in a few clinically well-defined cases [66], showing degeneration of the corticospinal tracts in the internal capsule, brainstem and spinal cord, while other studies have shown a significant loss of Betz cells in the motor cortex [67] without motor neuron loss in cranial nerve nuclei or the spinal cord. More recent immunohistochemistry studies [68] with TDP-43 staining do show inclusions in the hypoglossal nucleus and glial cells, though inclusions were sparse and inconsistent.

### 3.2. Neuroimaging in PLS

Neuroimaging in PLS has been limited and is usually undertaken in PLS as a subset of ALS cohorts. No clear diagnostic neuroimaging markers have been identified in PLS. MRI changes reported in PLS have included (i) atrophy specifically of the precentral gyrus [67], (ii) reduced N-acetyl aspartate/choline ratio over the primary motor cortex on MR spectroscopy, suggesting neuronal loss [69], (iii) reduced fluorodeoxyglucose (FDG) uptake over the precentral gyrus on positron emission tomography (PET) studies [70], suggesting reduced metabolism, (iv) increased quantitative FLAIR MRI intensity in both corticospinal tracts greater than the change noted in classical ALS [71] and (v) reduced fractional anisotropy and increased mean diffusivity in the corticospinal tracts and motor fibres of the corpus callosum [72], suggesting that motor fibres of the descending corticospinal tract and also more proximal segments are involved.

### 3.3. Neurophysiology in PLS

Cortical neurophysiology studies using transcranial magnetic stimulation reveal motor cortex inexcitability in PLS [73] with inability to elicit motor-evoked potentials in thenar intrinsic hand muscles at maximal stimulation or markedly prolonged motor-evoked potential latency [74,75]. The mechanism for this finding remains to be elucidated. However, in keeping with the previously described contribution to motor threshold on TMS testing [37,54], this finding may suggest altered corticomotorneuronal membrane properties, the loss of fast-conducting corticomotorneuronal fibres [50] or increased desynchronization of corticomotorneuronal volleys secondary to axonal loss [51].

## 4. Hereditary Spastic Paraplegia (HSP)

Hereditary spastic paraplegia (HSP) is a genetically diverse group of inherited disorders resulting in length-dependent dysfunction primarily of the corticospinal axons, though other spinal tracts such as the posterior columns may also be involved. They are now understood to be a family of genetic disorders causing dysfunction primarily of extremely long corticomotor axons due to a variety of genetic mutations, affecting pathways involved in the maintenance of axonal health and integrity [76]. The inherited axonopathy results in a syndrome of adult-onset, progressive upper motor neuron dysfunction in the legs or spastic paraplegia. The prevalence of the disease was reported as varying between 3–10/100,000 in various population-based studies [77], while the age of onset has a very wide range, between childhood and late adulthood. On average, the onset of HSP with unidentified genetic mutation is like that of PLS in a population-based study, making the two disorders difficult to distinguish at onset, while the commonest autosomal dominant variant of HSP has a younger age of onset compared with PLS [78].

Hereditary spastic paraplegia (HSP) has been categorised based on genetic inheritance into autosomal dominant, autosomal recessive or X-linked [77] primary categories. Autosomal dominant forms of HSP are most prevalent in Northern European and North American populations and 70–80% of these patients present with a pure HSP phenotype [76]. Autosomal recessive HSP has greater prevalence in populations with high consanguinity and is more complex in phenotype, including cerebellar signs, optic disc pallor and peripheral neuropathy [76,77]. X-linked HSP and HSP due to mitochondrial DNA mutations form a small proportion of total HSP cases and often have a complex phenotype. Newer gene mutations identified in HSP syndromes have also been associated with cortical changes in non-motor pathways [79,80], thereby extending the spectrum of pathogenesis in HSP beyond the corticospinal axon (Table 1).

### 4.1. Histopathology in HSP

Histopathology in HSP is limited due to the relatively normal lifespan of patients with pure HSP [76]. Neuropathology studies of the spinal cord in HSP [81] show a length-dependent loss of axons in the corticospinal and sensory tracts, with nerve fibre loss affecting both the large- and small-diameter axons. While axonal loss was widespread and symmetrical, its extent was length-dependent in the efferent motor and afferent sensory tracts. Spinal changes in HSP appear similar in complex phenotypes [82], while cortical changes have largely been case reports in isolated complicated forms of HSP. A variety of cortical changes, from thinning of the corpus callosum to changes in non-motor pathways and deep grey nuclei, have been reported [83], with immunohistochemistry revealing tau immunoreactive deposits in the limbic and neocortex with Lewy bodies identified in the substantia nigra [79]. While classical changes in the spinal cord appear to be a feature across the spectrum of HSP, cortical changes are variable across complicated HSPs, perhaps reflecting the functional spectrum of HSP genes involved in axonal transport.

### 4.2. Neuroimaging in HSP

Neuroimaging in HSP was initially a tool to exclude mimic causes of spastic paraplegia. Atrophy of the spinal cord without anteroposterior flattening measured most often at the cervical cord level is the most reported finding in pure and complicated forms of HSP [84]. Studies in less common recessive forms of HSP also report a signal change in the internal capsule and thinning of the corpus callosum [85], thereby refuting the previous clinical impression of HSP being a condition of corticospinal axons affecting the lower limbs alone. An attempt at correlation between cognitive dysfunction and global brain atrophy in pure and complicated forms of HSP revealed a weak correlation with clinical cognitive dysfunction [86], while other, larger series have identified white matter tract abnormality in the motor association area and cerebellum in a cohort of pure and complicated HSP phenotypes [87].

### 4.3. Neurophysiology in HSP

Cortical changes were not noted in a group of patients with the autosomal dominant (AD) genetic HSP when using the transcranial magnetic stimulation technique to probe motor cortex hyperexcitability [73] with motor-evoked potential (MEP) recording performed over the intrinsic hand muscles. Hyperexcitability of the motor cortex [88] was reported in HSP patients with specific gene mutations but not in large series. Motor-evoked potential latency was prolonged in 78% of all category HSP patients [89], which may supersede findings of individual studies to the contrary [90]. Changes in parameters such as the MEP latency and amplitude may reflect the corticospinal axon dysfunction in HSP. However, cortical hyperexcitability reflecting motor cortical changes has not been detected in AD HSP, despite the reported cerebral imaging changes [73].

## 5. Primary Progressive Multiple Sclerosis (PPMS)

Primary progressive multiple sclerosis (PPMS) has been studied under the category of inflammatory disorders of the central nervous system. PPMS is clinically an insidious-onset slowly progressive pure upper motor neuron disorder presenting with gait dysfunction [91]. Features that may differentiate primary progressive multiple sclerosis (PPMS) from HSP include brain and spinal cord lesions on MR imaging and the presence of oligoclonal bands in the CSF [92]. The majority of PPMS, like other variants of multiple sclerosis, show an association with the same haplotype of the major histocompatibility gene complex on chromosome 6 [93].

Interestingly, a case report of a patient with a clinical diagnosis of PPMS and a family history of Pelezaeus–Merzbacher disease revealed a point mutation in the proteolipid protein1 (PLP-1) gene [94]. PLP-1 codes for a membrane protein which forms a significant component of the protein in central nervous system (CNS) myelin and is also associated with spastic paraplegia type 2 (SPG2). This report highlights a pathophysiological link between the two reportedly disparate UMN disorders. One of these, Pelezaeus–Merzbacher disease, is a rare, inherited X-linked central nervous system dysmyelinating disorder akin to PPMS, while the other is an X-linked type of HSP with pure spastic phenotype (Table 1).

### 5.1. Histopathology in PPMS

The role of inflammatory cells causing pathological changes in the cerebral cortex and spinal cord in PPMS has been well described [95], with B and T cells, macrophages, activated microglia, dendritic cells and astrocytes all implicated in the pathology in PPMS. Interestingly, lesions in the white matter on MR imaging have varying pathology when compared to areas which appear as non-lesional areas on imaging [96]. Further, grey matter pathology, while less understood, was correlated with disability in MS in general [97]. Spinal cord pathology shows areas of partial demyelination in the spinal cord white matter, with evidence to suggest axonal thinning.

### 5.2. Neuroimaging in PPMS

Along with the wider subject of multiple sclerosis, PPMS has been extensively studied using neuroimaging modalities, and current diagnostic criteria require the presence of typical brain or spinal cord lesions for the diagnosis of PPMS [92]. The lesion load and contrast enhancement of lesions was found to be less in PPMS compared with the progressive variety of multiple sclerosis [98]. Several novel structural MRI techniques such as magnetization transfer ratio (MTR) and diffusion tensor MRI (DT MRI) revealed extensive changes in normal-appearing areas of brain grey and white matter in PPMS [99] and changes in the spinal cord suggesting atrophy. MR spectroscopy studies revealed evidence of axonal loss in lesions and non-lesional areas in PPMS detected as a reduction in N-acetyl aspartate (NAA), a marker of axonal integrity.

### 5.3. Neurophysiology in PPMS

Cortical excitability using transcranial magnetic stimulation technique [100] did reveal a progressive hyperexcitability in a subset of PPMS patients studied repeatedly over two years. However, reasonable diagnostic sensitivity and specificity of hyperexcitability or a large proportion of inexcitability of the motor cortex has not been described in PPMS in contrast to ALS [7] or PLS [73].

## 6. Conclusions

This review summarises the salient features of four adult-onset neurological diseases with progressive and prominent upper motor neuron signs, with emerging key differences in their pathophysiological mechanisms. An improved understanding of the upper motor neuron has been promoted by studies exploring the pathophysiology of these disorders.

Amyotrophic lateral sclerosis (ALS) is now recognized as a TDP-43 proteinopathy [22]. However, an exact understanding of the mechanism whereby TDP-43 becomes dysfunctional, aggregates and spreads within the cerebral cortex and the etiology behind rapidly progressive upper and lower motor involvement in classicals forms of this disorder remain to be elucidated. A consistent motor cortex phenomenon in ALS is of cortical hyperexcitability, which appears to be an early [8,47] and perhaps pathogenic mechanism in ALS [38,101] and may contribute to lower motor neuron dysfunction [6]. Newer studies suggest that cortical hyperexcitability in ALS may result from altered balance of intracortical inhibitory and excitatory circuits [43].

Primary lateral sclerosis (PLS) is also thought to be a TDP-43 proteinopathy [68], though it has unique characteristics [59]; specifically, slower progression and delayed involvement of the spinal motor neuron. Notably, PLS is most often associated with inexcitability of the motor cortex rather than hyperexcitability [73]. Better understanding of the difference in pathophysiology between ALS and PLS may promote understanding of TDP proteinopathy and, by extension, of other neurodegenerations associated with protein dysfunction.

Hereditary spastic paraplegia (HSP) is emerging as a disorder of a heterogenous family of genes which code for proteins involved in axonal transport, resulting in a length-dependent motor disorder affecting primarily the lower limbs [76,77]. Other central nervous system networks are also affected by the implicated gene dysfunction, resulting in complicated variants of HSP. Ongoing identification and understanding of genes associated with HSP have improved understanding of axonal transport in corticospinal neurons and of other associated cortical functions regulated by the dysfunctional genes and proteins. 

Primary progressive multiple sclerosis (PPMS) is increasingly being recognized as a neurodegenerative disorder, with neuroinflammation being the pathogenetic mechanism for neurodegeneration [91,95]. Notably, its pathology appears like classical relapsing remitting multiple sclerosis and the secondary progressive multiple sclerosis phenotype [95]. A better understanding of the insidious but relentless UMN dysfunction in PPMS as opposed to other phenotypes of multiple sclerosis might help clarify vulnerabilities of the UMN to such immune mechanisms. Progressive understanding of upper motor neuron function is, therefore, not an esoteric pursuit but a vital need for developing cures for UMN disorders or preventing pathophysiological mechanisms causing dysfunction of this complex system.

## Figures and Tables

**Table 1:** Comparison of the clinical, imaging, neurophysiology and histopathology changes in ALS, PLS, HSP and PPMS.

	ALS	PLS	HSP	PPMS
**Neurophysiology**	➢ Reduced intracortical inhibition, cortical hyperexcitability	➢ Increased motor cortex threshold with cortical inexcitability	➢ Increased motor potential conduction time in the legs	➢ Normal excitability of the motor cortex in the majority
**Neuroimaging**	➢ T2 hyperintensity of the corticospinal tract (CST) ➢ Reduced anisotropy and increased diffusivity of the corticospinal tracts	➢ Hypometabolism over the precentral gyrus on FDG-PET (stripe sign) ➢ Knife edge atrophy of the precentral gyrus ➢ Central callosal anisotropy on DTI	➢ T2 hyperintensity in the posterior limb of the internal capsule. ➢ Spinal cord atrophy ➢ Corpus callosum thinning	➢ White matter T2 and FLAIR hyperintense lesions in the brain and spinal cord.
**Histopathology**	➢ TDP-43 proteinopathy except in SOD-1 and FUS genetic ALS ➢ Spread of TDP-43 unrelated to clinical severity	➢ TDP-43 inclusions sparsely and inconsistently detected in cortical motorneurons, glial cells and hypoglossal nucleus.	➢ Length-dependent small and large fiber distal axonopathy of motor and sensory spinal tracts	➢ Widespread inflammatory white and grey matter infiltration with secondary neuronal and axonal loss
**Clinical**	➢ Average age of onset 65 y ➢ Male:female 1.2–1.4:1 ➢ Average survival 3 y ➢ UMN and LMN signs with varying phenotypes	➢ Average age of onset 50 y ➢ Male:female 2–4:1 ➢ Average survival 7.9 y ➢ Progressive pure UMN syndrome, commonly lower limb onset	➢ Average age of onset 40 y ➢ Male:female not known ➢ Normal lifespan ➢ Progressive lower limb onset UMN syndrome	➢ Average age of onset 40 y ➢ Male:female 1:1 ➢ Normal lifespan ➢ Progressive UMN syndrome presenting with gait dysfunction

Unique clinical, histopathology, neuroimaging and neurophysiology features that differentiate amyotrophic lateral sclerosis (ALS), primary lateral sclerosis (PLS), hereditary spastic paraplegia (HSP) and primary progressive multiple sclerosis (PPMS). Abbreviations: upper motor neuron (UMN); lower motor neuron (LMN); transactive response DNA-binding protein 43 (TDP-43); superoxide dismutase 1 (SOD-1); fused in sarcoma (FUS); corticospinal tract (CST); fluorodeoxyglucose-positron emission tomography (FDG-PET), diffusion tensor imaging (DTI); fluid-attenuated inversion recovery (FLAIR).
